# Response of Mycorrhizal ’Touriga Nacional‘ Variety Grapevines to High Temperatures Measured by Calorespirometry and Near-Infrared Spectroscopy

**DOI:** 10.3390/plants9111499

**Published:** 2020-11-05

**Authors:** Amaia Nogales, Hugo Ribeiro, Julio Nogales-Bueno, Lee D. Hansen, Elsa F. Gonçalves, João Lucas Coito, Ana Elisa Rato, Augusto Peixe, Wanda Viegas, Hélia Cardoso

**Affiliations:** 1 LEAF—Linking Landscape, Environment, Agriculture and Food. Instituto Superior de Agronomia, Universidade de Lisboa, Tapada da Ajuda, 1349-017 Lisboa, Portugal; elsagoncalves@isa.ulisboa.pt (E.F.G.); jlcoito@isa.ulisboa.pt (J.L.C.); wandaviegas@isa.ulisboa.pt (W.V.); 2 Departamento de Fitotecnia, MED-Instituto Mediterrâneo para a Agricultura, Ambiente e Desenvolvimento, Escola de Ciências e Tecnologia, Universidade de Évora, Pólo da Mitra, Ap. 94, 7006-554 Évora, Portugal; p1436@alunos.uevora.pt (H.R.); julionogales@us.es (J.N.-B.); aerato@uevora.pt (A.E.R.); apeixe@uevora.pt (A.P.); 3 Food Colour and Quality Laboratory, Department of Nutrition and Food Science, Facultad de Farmacia, Universidad de Sevilla, 41012 Sevilla, Spain; 4 Department of Chemistry and Biochemistry, Brigham Young University, Provo, UT 84602, USA; ldhansen@chem.byu.edu; 5 MED-Instituto Mediterrâneo para a Agricultura, Ambiente e Desenvolvimento, Instituto de Investigação e Formação Avançada, Universidade de Évora, Pólo da Mitra, Ap. 94, 7006-554 Évora, Portugal; hcardoso@uevora.pt

**Keywords:** *Vitis vinifera* L., stress tolerance, arbuscular mycorrhizal fungi, chlorophyll fluorescence, membrane permeability, relative chlorophyll content, stomatal conductance

## Abstract

Heat stress negatively affects several physiological and biochemical processes in grapevine plants. In this work, two new methods, calorespirometry, which has been used to determine temperature adaptation in plants, and near-infrared (NIR) spectroscopy, which has been used to determine several grapevine-related traits and to discriminate among varieties, were tested to evaluate grapevine response to high temperatures. ‘Touriga Nacional’ variety grapevines, inoculated or not with *Rhizoglomus irregulare* or *Funneliformis mosseae*, were used in this study. Calorespirometric parameters and NIR spectra, as well as other parameters commonly used to assess heat injury in plants, were measured before and after high temperature exposure. Growth rate and substrate carbon conversion efficiency, calculated from calorespirometric measurements, and stomatal conductance, were the most sensitive parameters for discriminating among high temperature responses of control and inoculated grapevines. The results revealed that, although this vine variety can adapt its physiology to temperatures up to 40 °C, inoculation with *R. irregulare* could additionally help to sustain its growth, especially after heat shocks. Therefore, the combination of calorespirometry together with gas exchange measurements is a promising strategy for screening grapevine heat tolerance under controlled conditions and has high potential to be implemented in initial phases of plant breeding programs.

## 1. Introduction

Grapevine (*Vitis vinifera* L.) is one of the most important crops worldwide with 7449 thousand hectares of cultivated area. Portugal has 192 thousand hectares of vineyards, and viticulture and wine production are among the most important socioeconomic activities of the Portuguese agricultural sector [1]⁠. However, due to climate change and global warming, grapevine yield and quality are likely to decrease in Southern Europe [2,3,4,5]⁠, especially in the southern and inner regions of Portugal that may already be at the limit of optimal conditions for high-quality wine production [6,7,8].

Transitory or constant high temperatures can lead to morpho-anatomical, physiological, and biochemical changes in plants, affecting plant growth and development with associated reductions in crop yield and fruit quality [9]. Direct negative effects of heat stress include protein denaturation and aggregation, and increased fluidity of membrane lipids, while indirect heat injuries include inactivation of enzymes in chloroplast and mitochondria, inhibition of protein synthesis, protein degradation, and loss of membrane integrity [10]. Such responses can adversely affect photosynthesis, respiration, and water relations, as well as modulate hormones and primary and secondary metabolite levels [9]. However, plants have evolved various mechanisms to respond to heat stress enabling them to grow and develop under elevated temperatures. In many cases, heat-tolerant species or varieties are characterized by higher photosynthetic rates, transpirational cooling, increased membrane thermostability and heat avoidance mechanisms including changes in leaf orientation [9,11,12]. Nonetheless, plant breeding for thermotolerance is hindered by the lack of knowledge on inter- and intravarietal variability concerning this trait, which is partly due to the lack of reliable screening tools to evaluate heat injury [13,14,15].

In agriculture, the use of heat tolerant cultivars or the promotion of heat tolerance by plant inoculation with selected endophytic microorganisms, such as arbuscular mycorrhizal fungi (AMF), may be a promising strategy to overcome the impact of high temperatures on important crops such as grapevines [8,16]. ‘Touriga Nacional’ is a highly appreciated red grapevine variety, used for Porto wine blends as well as for high quality table wine production [17]. It is well adapted to warm temperatures and has relatively high tolerance to excessive light stress, provided that there is no water scarcity [18,19]. When combined with a suitable rootstock, such as 1103 Paulsen (*Vitis berlandieri* x *Vitis rupestris*), which is in turn well adapted to drought and warm climates, it exerts greater plasticity of response to face new climatic challenges. 

Arbuscular mycorrhizal fungi establish symbioses with more than 80% of terrestrial plants [20] and provide multiple benefits to plants, including grapevines, that naturally form mycorrhizal symbioses under field conditions [21]. They improve nutrient uptake, especially P, as well as their tolerance to a variety of biotic and abiotic stress factors, such as drought and salinity [21]. Although plant inoculation with AMF has been claimed to promote plant tolerance to high temperatures in other crop plants [22,23,24], this strategy is still underexplored in viticulture [25]. The development of new screening techniques to evaluate heat tolerance will greatly contribute to identifying thermotolerant cultivars as well as to assess the potential benefits of AMF on plant heat stress tolerance.

To date, several physiological parameters have been used to assess heat injury and thermotolerance including net photosynthesis rate, photosynthetic O_2_ evolution rate, stomatal conductance [3,26,27,28,29,30], membrane thermostability (including electrolyte leakage and the content of thiobarbituric acid-reactive substances) [31,32,33,34], chlorophyll content [35,36,37], and chlorophyll fluorescence measured using the O-J-I-P test, which is indicative of photosystem II functioning [38]. However, these methods have several disadvantages [9,38,39,40]. 

Nogales et al. [41,42] demonstrated that calorespirometry could be a reliable screening tool for discriminating the best performing plant genotypes under temperature stress conditions and could be useful in the initial phases of plant breeding programs. Calorespirometric measurements as a function of temperature are conveniently made on rapidly growing tissues with a temperature-scanning calorimeter. Structural biomass formation rates (i.e., anabolic rates or specific growth rates) and carbon use efficiencies can be calculated from calorespirometric measurements of respiratory heat rates and CO_2_ production rates at selected temperatures in actively growing tissues [43], thus, enabling measurement of changes in plant respiratory characteristics over a temperature range, including stressful high temperatures [42]. Moreover, calorespirometric measurements also enable the identification of optimum and minimum temperatures for plant growth, which, in turn, distinguishes different phenotypes based on those characteristics, and hence, it can be an accurate technology for detecting differences in growth metabolism related to temperature [42,44,45,46,47,48]. To date, calorespirometry has been shown to be useful for identifying cold tolerant genotypes [42], and the present work tests the potential of calorespirometry for evaluating short- and long-term heat stress responses. 

Photosynthesis is a rate-limiting process in plant growth if carbon is the limiting resource, but plants can grow no faster than photosynthates can be processed by respiration. In contrast to measurements of photosynthetic properties, calorespirometry can directly determine specific growth rates [44], whether the limitation is due to photosynthate, water, nutrient availability, or is inherent in the genetic background [49,50]. Any environmental condition that affects growth rates (e.g., temperature stress) can be quantified by calorespirometric measurements of growth rate as a function of the stress. Catabolism of photosynthates produces CO_2_, ATP, and NADH. Anabolic reactions are coupled to catabolism and use ATP and NADH for the synthesis of new tissue and for maintenance [50]. Anabolism also produces CO_2_ from reduction in photosynthate, but because anabolism produces very little or no heat [51], the rate of CO_2_ production from catabolism can be distinguished from the CO_2_ produced by anabolism through calorespirometric measurements. The CO_2_ rate from anabolism is directly proportional to the growth rate, and therefore can be estimated from calorespirometric measurements. 

Spectroscopy and hyperspectral imaging have also arisen in the last few years as important nondestructive methods to acquire relevant crop spectral information for phenotype characterization [52]. Among these methods, near-infrared (NIR) spectroscopy is widely used for studying different agronomic characteristics of grapevines [53], for example, grape composition [54], total phenolic compounds, anthocyanin profiles, and grape maturity [55,56,57], as well as grapevine petiole nutrient concentration [58], identification of grape berry sunburn symptoms [59], plant water status [60,61], and grapevine varietal discrimination [52,62]. Hence, NIR spectroscopy is a promising tool for precision viticulture [52] and a potentially attractive technology for screening new grapevine clones with enhanced thermotolerance in plant breeding programs.

Therefore, the objectives of this study were (1) to assess if AMF inoculation of ‘Touriga Nacional’ plants grafted onto 1103 Paulsen rootstock additionally increases their tolerance to high temperatures and (2) to evaluate the effectiveness of calorespirometry and NIR spectroscopy for evaluating heat stress response in grapevines.

## 2. Results

### 2.1. ‘Touriga Nacional’ Variety Vines Grafted onto 1103 Paulsen Rootstock were Naturally Colonized by Mycorrhizal Fungi

**Figure 1:**
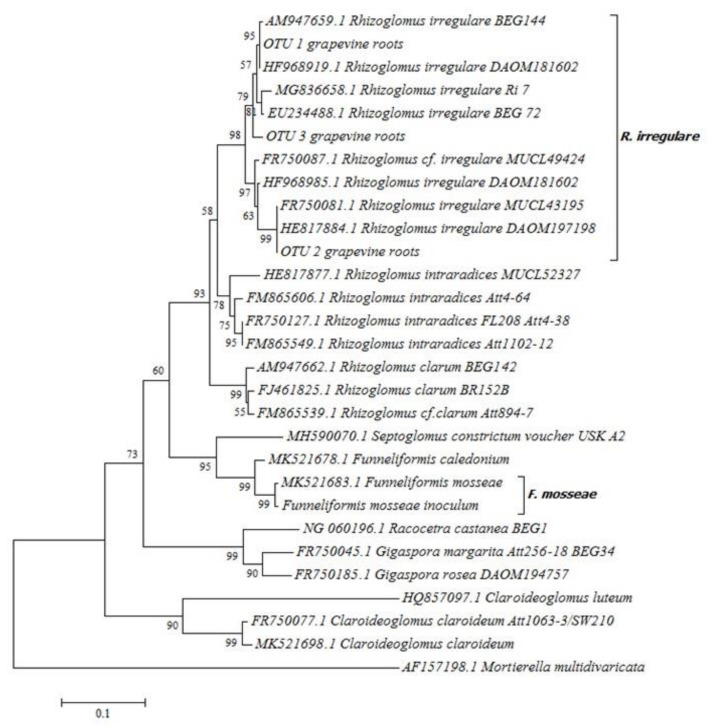
Neighbor-joining tree of nuclear large subunit (LSU) ribosomal RNA gene sequence. The tree shows the phylogenetic relationships among the tree operational taxonomic units (OTUs) identified in original grapevine root samples proceeding from the nursery, the two arbuscular mycorrhizal fungi (AMF) present in the inocula used in the experiment, and other representative species and isolates of Glomeromycota phylum. The optimal tree with the sum of branch length = 1.93277612 is shown. The percentage of replicate trees in which the associated taxa clustered together in the bootstrap test (1000 replicates) are shown next to the branches [65]. The tree is drawn to scale, with branch lengths in the same units as those of the evolutionary distances used to infer the phylogenetic tree. The evolutionary distances were computed using the Tamura–Nei method [66] and are in the units of the number of base substitutions per site. All positions containing gaps and missing data were eliminated. Evolutionary analyses were conducted in MEGA7 [67]. Taxon names used in the neighbor-joining tree were adapted according to the latest nomenclature of AMF published at Mycobank.

The rooted ‘Touriga Nacional’ plants grafted onto 1103 Paulsen rootstock that were used in this study, were obtained from a plant nursery, and were spontaneously colonized by native AMF. Thirty fungal DNA sequences were obtained after root DNA isolation and amplification with specific primers [63,64], from which six were identified as quimeras and deleted from the analysis. The remaining sequences were grouped into three operational taxonomic units (OTUs). Phylogenetic analysis revealed that the three OTUs belonged to the *Rhizoglomus irregulare* species (Figure 1).

Three and 10 months after plant inoculation with *Rhizoglomus irregulare* (Błaszk., Wubet, Renker & Buscot) Sieverd., G.A. Silva & Oehl or with *Funneliformis mosseae* (T.H. Nicolson & Gerd.) C. Walker & A. Schüßler, all plants were colonized by AMF (Table 1). Non-inoculated grapevines were also colonized, indicating the presence of native AMF. In fact, in both time points, those plants presented significantly higher colonization rates than plants inoculated with *R. irregulare* (Table 1). Considering that greenhouse-grown grapevines usually show root colonization rates between 66 and 91% when symbiosis is well developed and functional [68], at sprouting time of the second growing year (10 months after inoculation), plants were considered to have a well-established mycorrhizal symbiosis.

#### 2.2. Grapevine Vegetative Growth Parameters were Influenced by Mycorrhizal Inoculation in the First Growing Season

**Figure 2:**
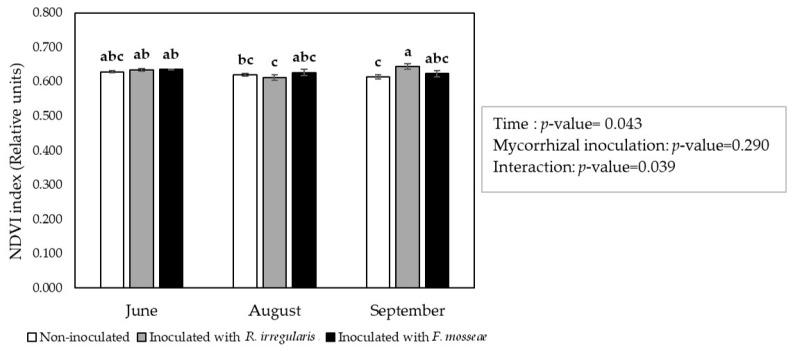
Normalized difference vegetation index in ‘Touriga Nacional’ variety vine plants grafted onto 1103 Paulsen rootstock measured along the first growing season. Bars indicate the average value (*n* = 5) ± standard error. Different letters indicate significant differences in group means according to pairwise multiple comparisons conducted using the simulation method of Edwards and Berry [70]. Values within the box indicate the *p-*value for the significance of the main factors and their interaction. Significant effect, *p*-value < 0.05.

The normalized difference vegetation index (NDVI), which is an indirect measurement of plant vigor, chlorophyll content, and N and P uptake [69], was monitored during the first growing season. A significant interaction was detected among *Time* and *Mycorrhizal inoculation* factors. In June and August, three and five months after inoculation, no significant differences were observed in this parameter among the three inoculation treatments. However, in September, by the end of the first growing season, *R. irregulare-*inoculated plants showed significantly higher values than non-inoculated plants, and *F. mosseae*-inoculated plants showed intermediate values (Figure 2).

**Figure 3:**
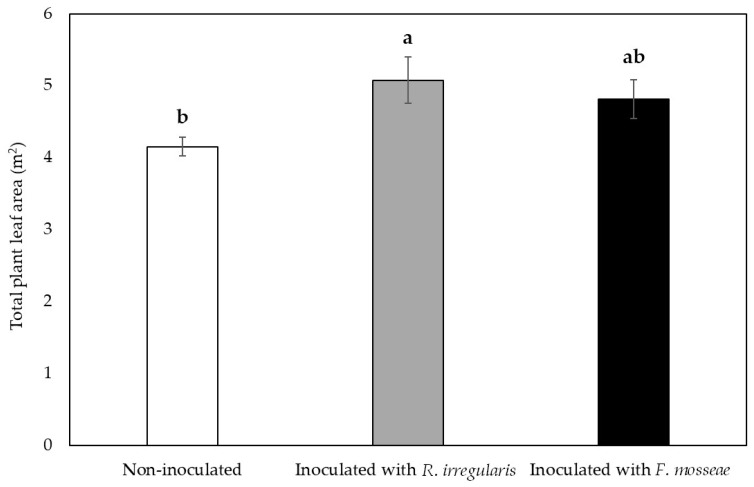
Total leaf area per plant in ‘Touriga Nacional’ variety vine plants grafted onto 1103 Paulsen rootstock. Bars indicate the average value (*n* = 5) ± standard error. Different letters indicate significant differences in group means according to Duncan’s *a posteriori* test. Significant effect, *p*-value < 0.05.

Concerning total plant leaf area, in August, when maximum plant growth was considered to be achieved, *R. irregulare*-inoculated plants showed significantly higher values than non-inoculated plants, while *F. mosseae-*inoculated plants showed again intermediate values (Figure 3).

#### 2.3. ‘Touriga Nacional’ Variety Grapevines Adapted Their Physiology to Elevated Temperatures

Physiological parameters evaluated in the second growing year, before and after plant exposure to elevated temperatures (five days at 40/35° day/night regime, 16 h photoperiod), indicated a significant main effect of *Long-term heat stress exposure* factor on relative electrolyte leakage (EL), stomatal conductance (g_s_), and relative chlorophyll content (ChlC) of mature leaves, but not on the ratio of variable fluorescence to maximum fluorescence (Fv/Fm) (Table 2).

**Figure 4:**
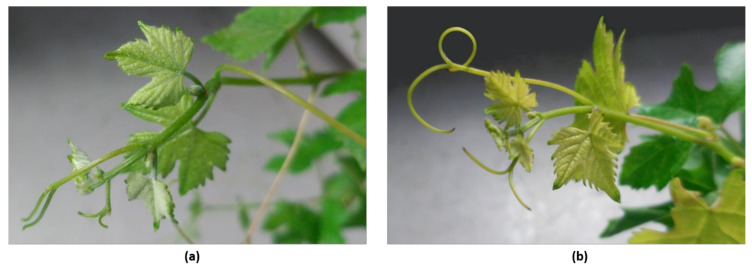
Apical leaves of a ‘Touriga Nacional’ grapevine plant. (**a**) Before plant exposure to 5 days of heat stress; (**b**) After plant exposure to heat stress for 5 days.

While EL showed an overall decrease in its values after five days exposure to 40/35 °C day/night temperatures, ChlC presented an overall increase after heat stress exposure (Table 3). However, multiple comparison tests did not show statistical differences among the different experimental group means in EL and in ChlC (Table 3). In contrast to mature leaves, the new leaves developed at 40/35 °C day/night presented chlorosis symptoms in all plants, where the entire leaf area lost its typical color (Figure 4).

No main significant effects were detected for *Mycorrhizal inoculation* factor in EL, ChlC, and Fv/Fm. However, in g_s,_ both, *Long-term heat stress exposure* and *Mycorrhizal inoculation* factors had a significant effect (Table 2), and no significant interaction was detected among both factors. After five days of plant exposure to high temperatures, g_s_ significantly increased in *R. irregulare-* and *F. mosseae-*inoculated plants (293% and 192% increase, respectively), while no statistical differences were observed in non-inoculated plants before and after heat stress exposure (Table 3). 

#### 2.4. Calorespirometric Measurements Detected Short- and Long-Term Heat Stress Responses

Calorespirometric measurements were done on the first expanded leaf of inoculated and non-inoculated grapevine plants before and after plant exposure to a short-term heat stress (heat shock) for two hours at 40 °C, and to a long-term heat stress of five days at 40/35 °C day/night. Metabolic heat rate (R_q_) and respiratory rate (CO_2_ production rate, R_co2_) were monitored in dark-adapted leaves, and specific growth rate or structural biomass formation rate (R_biomass_) and carbon use efficiency (Ɛ) were calculated from R_q_ and R_co2_ data. No significant interactions among factors were found. *Short-term heat stress exposure* factor had significant main effects on all parameters except on R_co2,_ while *Long-term heat stress exposure* factor had significant main effects on all four calorespirometric parameters (Table 4). Overall, *Mycorrhizal inoculation* factor did not have a significant main effect, but when data were analyzed by a multiple comparison test, differences were detected among group means of the different mycorrhizal and temperature treatment combinations (Table 4).

**Figure 5:**
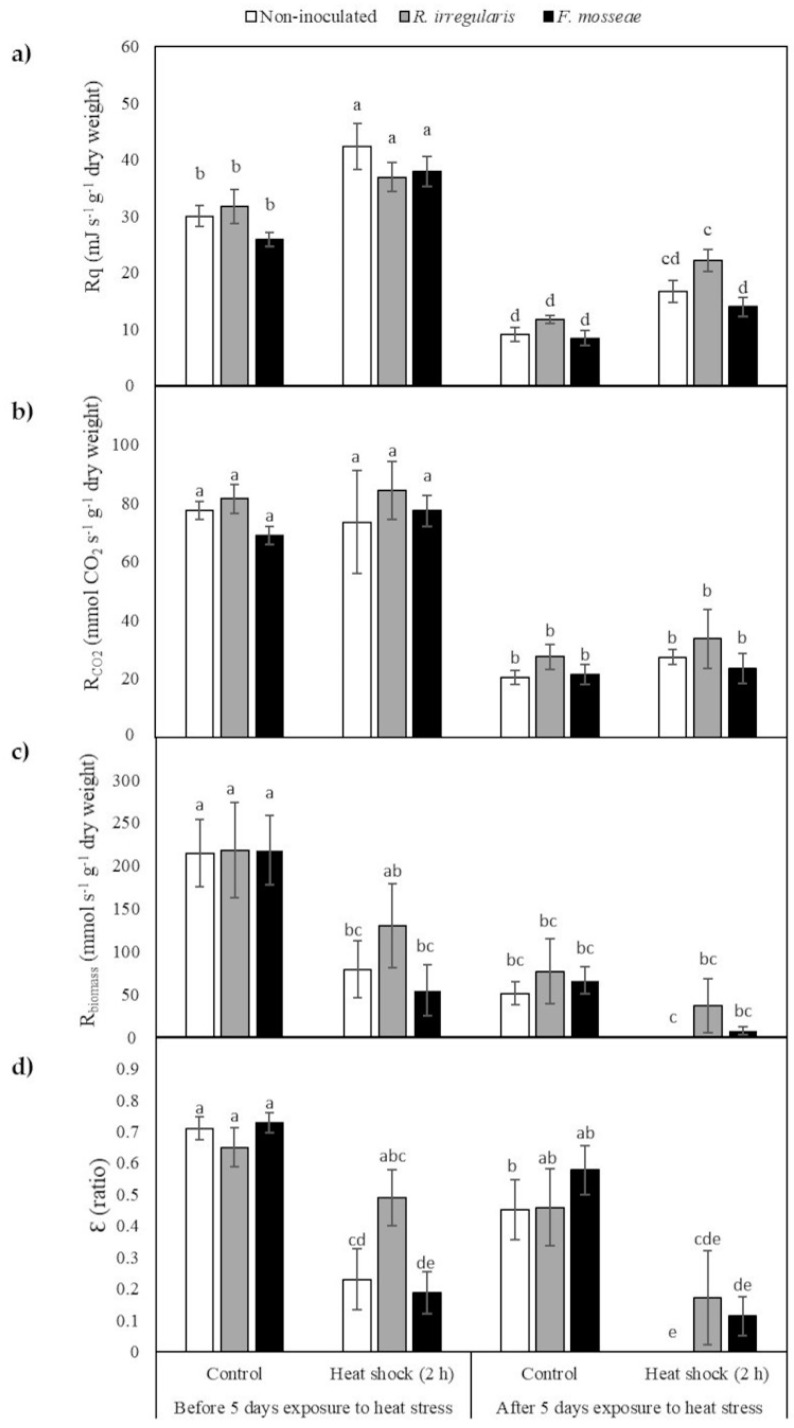
Calorespirometric parameters. (**a**) Metabolic heat rate, R_q_; (**b**) CO_2_ production rate, R_co2_; (**c**) Structural biomass formation rate, R_biomass_; (**d**) Carbon use efficiency, Ɛ. Bars represent the average of five samples ± standard error. Different letters indicate significant differences according to pairwise multiple comparisons conducted using the simulation method of Edwards and Berry [70]. Significant effect, *p*-value < 0.05.

When a two-hour heat shock was applied to plants growing in non-stressful temperatures (25/18 °C day/night), a significant increase in R_q_ was observed, while R_co2_ values remained unchanged in the three inoculation treatments (Figure 5a,b). In addition, a decrease in R_biomass_ and Ɛ was observed in non-inoculated and *F. mosseae* -inoculated plants (60% and 82% decrease in R_biomass_, respectively, and 68% and 75% in Ɛ, respectively). *Rhizoglomus irregulare*-inoculated plants had similar values to the those observed before the heat shock (Figure 5c,d). However, in plants exposed to a five-day elevated temperature regime (long-term heat stress), when an additional two-hour heat shock was applied, a significant decrease was observed in all calorespirometric parameters as compared with the plants that had not been exposed to any kind of heat stress treatment. The decrease in R_biomass_ and Ɛ was largest in non-inoculated plants, in which growth completely stopped, followed by *F. mosseae*-inoculated plants, that presented a decrease in growth of 97% and 85% in R_biomass_ and Ɛ, respectively, and by *R. irregulare*-inoculated plants, with a decrease in growth of 91% and 80% in R_biomass_ and Ɛ, respectively (Figure 5c,d).

#### 2.5. Near-Infrared Spectroscopy

**Figure 6:**
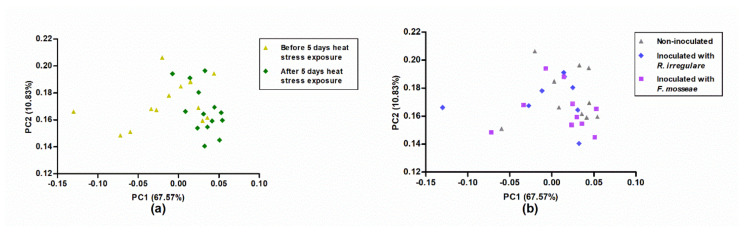
Score plot of plant samples in the plane defined by principal components 1 (PC1) and 2 (PC2) considering (**a**) exposure to elevated temperatures, before and after five days heat stress exposure, or (**b**) different mycorrhizal inoculation treatments, i.e., non-inoculated, inoculated with *R. irregulare* or inoculated with *F. mosseae*.

The principal component analysis (PCA) performed using NIR spectra collected in mature grapevine leaves, before and after five days plant exposure to 40/35 °C day/night temperatures, showed that 99% of the NIR spectral variability could be explained by 16 principal components (PCs). Moreover, for each spectral sample, through PCA, the Mahalanobis distances (H) were obtained from the mean spectrum of the entire sample set in this hyperspace. Samples were ranked in order of their H values. No spectral outliers were detected by the application of the H > 3 criterion. Figure 6 plots the scores of the samples in the plane made up of the first and second PCs. These PCs described 67% (PC1) and 11% (PC2) of the variability in the spectral data. In this plot, differences related to long-term heat stress exposure (Figure 6a) or to the mycorrhizal inoculation treatment (Figure 6b) were not apparent, since samples overlapped.

Next, quantitative calibrations were performed by a modified partial least squares (MPLS) regression using as reference variables EL, ChlC, g_s_, Fv/Fm, R_q_, R_co2_, R_biomass_, and Ɛ. Table 5 shows the main statistical parameter estimates obtained for each calibration equation. The robustness of the method was checked by the leave-one-out validation method. The lowest standard error of cross-validation (SECV) was observed for the Fv/Fm variable (1.81%), indicating that the model based on NIR and Fv/Fm data was the most robust one, followed by the ones based on EL, ChlC, and Ɛ, with SECV values of 8.43%, 13.37%, and 20.78%, respectively. For the other variables, the models were not considered suitable. Internal validations were also carried out using samples that belonged to the calibration group, excluding the corresponding T outliers in each case and predicting the reference values by the developed models. The standard errors of prediction (SEP) in internal validation were obtained as a result (Table 5).

**Figure 7:**
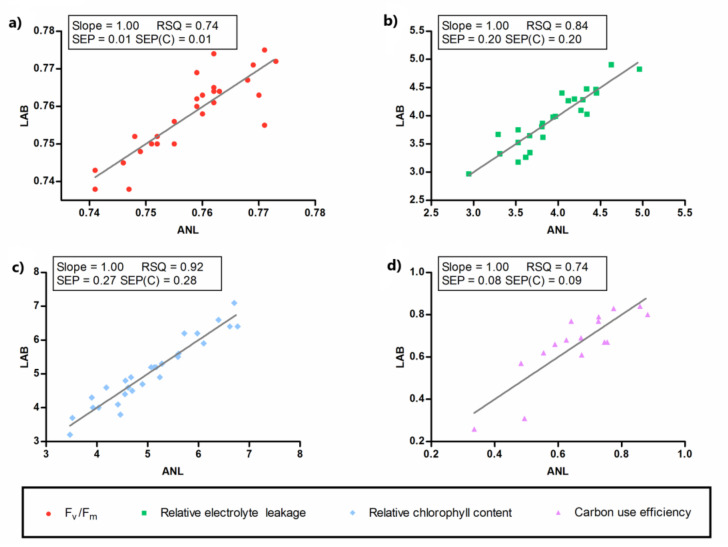
Comparison between the predicted data (ANL) and the data obtained experimentally (LAB) for the reference variables. (**a**) Fv/Fm; (**b**) Relative electrolyte leakage; (**c**) Relative chlorophyll content; (**d**) Carbon use efficiency. RSQ, coefficient of determination; SEP, standard error of prediction (internal validation); SEP(C), standard error of prediction corrected for the bias.

Figure 7 shows a comparison between the predicted data and the data obtained experimentally for Fv/Fm, EL, ChlC, and Ɛ. The slope, coefficient of determination (RSQ), SEP, and standard error of prediction corrected for the bias (SEP(C)) obtained in the internal validation for the predictions of these variables are also presented in Figure 7. These parameters confirm the good correlation between the predicted and the experimental data, and therefore, the validity of the obtained models for the screening of Fv/Fm, EL, ChlC, and Ɛ. 

## 3. Discussion

In this study, mycorrhizal inoculation of grafted ‘Touriga Nacional’ vines that were already colonized with native AMF from the nursery, led to a decrease in root colonization rates, especially in plants inoculated with *R. irregulare* (Table 1), which may reflect an intraradical competition effect for host resources and space [72,73,74,75,76]. However, this was not translated into a plant performance reduction. On the contrary, plants benefited from the inoculation with this AMF in terms of vigor during the first growing season (Figure 2 and Figure 3) as compared with the non-inoculated plants. 

Although AMF are known to generally improve host plant’s stress tolerance [77], it is known that some species, and even different isolates within the same species, can differentially influence their growth and physiological responses under abiotic stress conditions [78,79,80,81], and therefore some AMF are better suited than others for certain environments. With the aim of detecting the potential benefits of plant inoculation with two different AMF species in a heat tolerant grapevine variety exposed to elevated temperatures, in the present work, several methods were compared. Leaf chlorophyll content and electrolyte leakage have traditionally been used as indirect measures of heat stress tolerance in diverse plant species [33,82,83,84,85,86], as high stressful temperatures commonly lead to a decrease in the amount of photosynthetic pigments [85,87,88] and also affect the integrity and functions of biological membranes, enhancing their permeability and leading to cell electrolyte leakage [9]. However, exposure of ‘Touriga Nacional’ grapevine plants to high temperatures for five days led to a general increase in ChlC and a decrease in EL in mature leaves (Table 2 and Table 3). Moreover, no changes were observed in the Fv/Fm ratio, which has been claimed to be more suitable than electrolyte leakage in grapevines to assess heat injury [38]. The fact that mature leaves of the ‘Touriga Nacional’ plants did not show stress symptoms after five days exposure to elevated temperatures and exhibited opposite responses to those expected, indicates that plants were able to acclimate to the temperature regime used (40/35 °C day/night). The stress regime applied by Xu et al. [38] that led to an increase in electrolyte leakage and a decrease in Fv/Fm ratio was 47 °C for 50 min, which was the critical temperature for irreversible heat injury in grapevine leaves. In another study conducted on grapevines with the same temperature regime as the one applied in our study (40/35 °C day/night, 16 h photoperiod), Fv/Fm showed a decrease after the heat stress treatment, but in that case, Fv/Fm measurements were performed in the most recent expanded leaves, while our measurements were performed in mature leaves [89]. Because young leaves have usually exhibited greater physiological damage from heat stress than mature leaves [90], the decrease in Fv/Fm may have been observed in young leaves but not in the mature ones under the temperature regime applied in our experiment, although differences in cultivar sensitivity to high temperatures could also explain the discrepancy between the results of Kadir et al. [89] and our experiment.

A high temperature acclimation process could also explain the decrease in EL and the increase in ChlC and g_s_. Increasing membrane lipid saturation for achieving a higher cellular membrane rigidity is a major strategy by which plants adapt to high temperatures [9,91,92,93,94]. Several authors have observed that plant genotypes with high heat tolerance presented higher levels of saturated fatty acids and lower electrolyte leakage than the more heat-sensitive genotypes after exposure to supra-optimal temperatures [95]. Therefore, in our plants, the decrease in EL in mature leaves exposed to high temperatures may indicate a change in membrane composition to adapt to high temperatures. 

In addition, chloroplasts also play a key role in physiological adaptive processes and metabolic reprogramming to respond to heat stress [96]. Hence, the increase in ChlC in mature leaves may be related to metabolic adaptations of the photosynthetic apparatus for optimizing plant growth and development under stress conditions. However, plant responses and adaptation to elevated temperatures needs to be much better understood to confirm this hypothesis. 

EL nor ChlC were both not affected by the different mycorrhizal inoculation treatments, which suggests that neither root mycorrhizal communities differed in their capacity to modulate these plant characteristics, or that ‘Touriga Nacional’ plants had already well developed mechanisms to adapt these processes to elevated temperatures and did not benefit from additional AMF inoculations. 

As previously mentioned, the temperature treatment used in the present study, induced an overall increase in g_s_ in mature leaves (Table 2 and Table 3). Under high soil moisture and air humidity conditions, other authors have also observed a g_s_ increase at high temperatures [97,98], suggesting that this enabled higher evaporative cooling and photosynthetic rates in plants, thereby helping them to survive short heat waves when there was enough water in the soil [98]. As the ability to sustain leaf gas exchange under heat stress has a direct relationship with heat tolerance, the observed increase in g_s_ in mature leaves may indicate an acclimation response to high temperatures [9]. In fact, other authors have previously reported that the ‘Touriga Nacional’ variety had high capacity to dissipate heat through evaporative cooling, concluding that this variety was well adapted to warm climate conditions [18]. In this study, as the increase in g_s_ under sustained high temperatures was higher in *R. irregulare*- and *F. mosseae*-inoculated grapevines than in non-inoculated plants (Table 3), this indicated that AMF inoculation improved plant capacity to regulate their leaf gas exchange at elevated temperatures, and therefore enhanced acclimation to these conditions. 

Although high temperatures did not lead to apparent damage in mature grapevine leaves, chlorosis was observed in newly developed leaves after a five-day exposure to elevated temperatures (Figure 4), indicating their higher susceptibility to the temperature regime applied in this study. Calorespirometric measurements on young leaves agreed with these observations, as after five days of high temperature exposure, R_q_, R_CO2_, R_biomass_, and Ɛ all decreased (Figure 5). A decrease in dark respiration and growth is a common response to heat stress [9] as it alters microtubular cytoskeleton organization with negative consequences on cell differentiation, elongation, and expansion [99,100].

Criddle et al. [44] were the first to demonstrate the accuracy of calorespirometric-based predictions by comparing predicted growth rates with measured growth rate data on tomato and cabbage leaves at different temperatures. Since then, many other authors have shown that, in green tissues, biomass growth rate was correlated with the R_biomass_ calculated from R_q_ and R_CO2_ [50,101,102]. Therefore, a decrease in R_biomass_ detected in young leaves of plants exposed to high temperatures is indicative of an overall growth rate decrease. Moreover, the calorespirometric method clearly showed differences in both the long-term heat stress response and the short-term stress response. After a two-hour heat shock at 40 °C, R_biomass_ and Ɛ showed a general decrease in all plants, except in the plants inoculated with *R. irregularis* (Figure 5), indicating that these plants were able to sustain both R_biomass_ and Ɛ at similar levels to those observed prior to the heat shock, and thus that they had higher tolerance to short-term exposure to elevated temperatures. After five-day exposure to high temperatures, an additional heat shock of two hours also led to a reduction in R_biomass_ and Ɛ values, but in this case, the decrease with respect to non-stressed plants growing at 25/18 °C (before any kind of stress was applied to them) was significant in all inoculation treatment plants, although the highest decrease was observed in non-inoculated plants, in which growth was completely (Figure 5c,d).

Overall, these results suggest that ‘Touriga Nacional’ vines grafted onto 1103 Paulsen rootstock were relatively tolerant to elevated temperatures as they adapted their physiology to better respond to them, as demonstrated by the observed increase in ChlC and g_s_, the decrease in EL, and the lack of changes in Fv/Fm, although young tissues stopped their growth and showed chlorosis symptoms. Of all parameters, only g_s_, R_biomass_, and Ɛ showed differences among the mycorrhizal inoculation treatments related to high temperature exposure. As ‘Touriga Nacional’ vines are already well suited to efficiently respond to warm temperatures [19,103], differences resulting from the effect of distinct root AMF species composition in plant growth and physiology may not be as evident as the differences that may result in a less tolerant cultivar. In fact, several studies have shown that susceptible cultivars tended to benefit more from AMF colonization than tolerant cultivars under stress conditions [104,105,106,107]. However, considering that the increase in g_s_ was higher in *R. irregulare-* and *F. mosseae*-inoculated plants, but that *R. irregulare* was the most effective AMF in sustaining R_biomass_ and Ɛ under heat stress, especially under short heat shocks, it seems that inoculation of ‘Touriga Nacional’ vines grafted onto 1103 Paulsen rootstock can additionally help these plants to better withstand high temperatures, even if plants are already colonized with native AMF, provided that they are under non-limiting soil water conditions. 

Near-infrared spectra collected in adult grapevine leaves did not show any pattern related to heat stress or to mycorrhizal inoculation treatments, as shown in Figure 6. However, suitable models could be created from NIR data to predict Fv/Fm, EL, ChlC, and Ɛ (Table 5 and Figure 7), which, with the exception of Fv/Fm, were all affected by high temperatures (Table 2 and Table 4). Although these findings may seem contradictory, the explanation rests on the fact that PCA is an unsupervised pattern recognition technique, whereas MPLS is a supervised one. This means that the new variables created by PCA, the principal components (PCs), collect the general variability of the spectral matrix, while the new variables created in MPLS (PLS factors) only include the variability of the spectral matrix linked to the variability in reference parameters. Therefore, if the spectral variability associated to the temperature regimes applied has a small importance in the spectral matrix, it is possible that PCA does not identify it, whereas MPLS does.

This is the first study where NIR spectroscopy has been applied to the prediction of EL, ChlC, and Ɛ. In this study, the statistical parameters reported for SECV, SEP, SLOPE, and RSQ, were similar to the ones previously obtained in other studies where NIR-based technology was used in oenology and viticulture [55,56,57,61,62]. This indicated that NIR spectral measurements could be suitable for predicting all four variables (Fv/Fm, EL, ChlC, and Ɛ) from a single spectral measurement. The potential of NIR technology for grapevine phenotyping was highlighted by Gutierrez et al. [52] as an attractive and promising tool for precision viticulture, but the combination of calorespirometry with g_s_ could greatly increase its power for accurately phenotyping heat stress tolerance, as well as other complex abiotic stress tolerance traits. Therefore, the results obtained encourage testing these methods on a larger number of samples to enhance their robustness for the prediction of these parameters.

## 4. Material and Methods

### 4.1. Biological Material

*Vitis vinifera* L. ‘Touriga Nacional’ variety plants grafted onto 1103 Paulsen rootstock were obtained from VitiOeste plant nursery (Portugal). The rootstock has high rooting capacity [18] and it is widely used in Portugal to graft ‘Touriga Nacional’ variety grapevines. Roots were washed with tap water, cut to 2 cm, and three samples were collected for determining by molecular techniques the presence and identity of native AMF species colonizing them.

Plants were planted in 600 mL forest pot containers filled with an autoclave-sterilized (40 min at 120 °C in two consecutive days) substrate mixture of Sphagnum peat/perlite (2:1, v:v). The pH of the substrate was adjusted to 7.5 with CaCO_3_. At planting time, grapevines were inoculated with AMF; five plants were inoculated with 10 g of *Rhizoglomus irregulare* BEG 72 isolate inoculum (~100 infective propagules per g of carrier material) kindly provided by the Agrifood Institute of Research and Technology (IRTA, Barcelona, Spain) and another five plants were inoculated with 20 g of *Funneliformis mosseae* inoculum (~55 infective propagules per g) obtained from Symbiom Company (Checz Republic). Inoculation was done by placing the inoculum in the planting hole in contact with roots. Another five plants were left as non-inoculated control plants, where 20 g of the carrier material (devoid of mycorrhizal propagules) was added [108]. 

### 4.2. Molecular Identification of Arbuscular Mycorrhizal Fungi

To identify mycorrhizal fungal species/isolates present in grapevine roots from the nursery, root samples were collected from three different plants. DNA was extracted using a PowerSoil^®^ DNA Isolation Kit (Mo-Bio Laboratories, Inc., Carlsbad, CA, USA), following manufacturer’s instructions. Additionally, root fragments from *F. mosseae* inoculum were collected and DNA was extracted following the same procedure. Then, ribosomal RNA gene sequences including the partial large subunit region (LSU region) were amplified through a nested PCR using the primers LR1 and NDL22 [63] in the first reaction and FLR3 and FLR4 in the second reaction [64]. The PCR product of around 380 bp was cloned onto a pGEM^®^-T Easy vector (Promega, Madison, WI, USA) and sequenced by Sanger method (STABvida-Investigação e Serviços em Ciências Biológicas, Lda., Lisboa, Portugal). Ten recombinant bacterial clones per grapevine root sample and four clones of *F. mosseae* inoculum were sequenced.

Chimeric sequences were identified with Mothur program [109] using the UCHIME algorithm [110], and subsequently removed. The rest of the sequences were clustered at 97% similarity and assigned to operational taxonomic units (OTUs). One representative sequence from each OTU, the consensus sequence of the four *F. mosseae* clones obtained from the inoculum, and other partial sequences of the LSU region of different AMF species and isolates publicly available at the NCBI database (https://www.ncbi.nlm.nih.gov/) were aligned in ClustalW using the software MEGA V7.0 [67]. Partial LSU sequence of the AMF present in the other inoculum, *R. irregulare* BEG 72, formerly known as *Glomus intraradices* [111], also publicly available, was retrieved from this same database (Accession number EU234488.1).

A phylogenetic tree was constructed with genetic distances inferred by the neighbor-joining method [112]. *Mortierella multidivaricata* (R.K.Benj.) Benny & M.Blackw was used as an outgroup and the bootstrap consensus tree was inferred from 1000 replicates.

### 4.3. Plant Growth Conditions and High Temperature Treatment

Grapevine plants were grown under greenhouse conditions at the Instituto Superior de Agronomia (Higher Institute of Agronomy) University of Lisbon (Portugal) for three months. During this time, they were pruned to leave only two shoots per plant. One gram of slow release NPK fertilizer (Bayfolan Multi, Bayer, Bologna, Italia) was applied in each container.

After three months, plants were transplanted to containers filled with 4 kg of sterile sandy soil, and then maintained at open air conditions during a complete growing season. Watering and fertilization regimes were the same as in [108].

In the second growing year, before leaf sprouting, plants were transported to the Institute of Agricultural and Environmental Sciences (MED, Universidade de Évora, Évora, Portugal), and kept under greenhouse conditions. Each plant was fertilized with 500 mg of N and K and 50 mg of P, and daily watered with 300 mL tap water.

Three weeks after the grapevines sprouted new leaves, plants were moved to a growth chamber with controlled light and temperature conditions (25/18 °C day/night, 16 h photoperiod, 60% relative humidity, photon flux density of 300 µmol m^−2^ s^−1^). After five days growth under these conditions, the planned measurements were made on all plants. Then, a long-term heat stress treatment was applied during another five days by exposing the plants to 40/35 °C day/night temperatures with 16 h photoperiod, 60% relative humidity, and photon flux density of 300 µmol m^−2^ s^−1^, after which the planned measurements were repeated on all plants. Therefore, each plant analyzed under non-stressful conditions was its own control for studying heat stress response, and statistical analysis was performed accordingly (see below).

In all plant growth conditions (in the greenhouse, in open air, and in the growth chamber) the place of each plant was randomly assigned.

### 4.4. Determination of Mycorrhizal Colonization Rate

Three months after inoculation, when plants were transplanted to pots filled with sterile sandy soil, and ten months after inoculation, before plant sprouting at the beginning of the second growing year, root samples of three and five plants were collected, respectively, to estimate mycorrhizal root colonization. As the heat stress duration of the experiment (five days) was assumed to be too short for significant changes in root mycorrhizal colonization rates, plant colonization was not checked again after this period.

Roots were stained with 0.05% Trypan blue in lactic acid, following the protocols of [113] and [114]. The root colonization rate was determined using the gridline intersect method under a 40× optical microscope Olympus [115].

### 4.5. Plant Growth and Physiology Measurements

In the first growing year, normalized difference vegetation index (NDVI) was measured with a NDVI PlantPen NDVI 300 (PSI) portable device throughout the summer (June, August, September). In August, when total plant leaf area was expected to be maximum, measurements were done to estimate this variable [116].

In the second growing year, stomatal conductance (g_s_), Fv/Fm ratio (the ratio of variable fluorescence to maximum fluorescence), relative chlorophyll content (ChlC), and relative electrolyte leakage (EL) were measured in two, mature, fully expanded leaves (approximately 6 cm diameter) from each of the five plants of each mycorrhizal treatment (non-inoculated, inoculated with *R. irregulare,* and inoculated with *F. mosseae*) before and after plant exposure to elevated temperatures for five days. Replicate measurements were done on each leaf and the average was used in the statistical analysis. Stomatal conductance was measured with a porometer AP4 from Delta-T (UK) between 1:00 and 1:30 p.m. The ratio Fv/Fm was measured with a chlorophyll fluorometer OS-30p+ from Opti-Sciences (USA) in previously dark-adapted leaves. Relative chlorophyll content was measured with a Chlorophyll Content meter CL-01 from Hansatech Instruments (UK).

Determination of EL, which is an indirect estimation of the integrity of cellular membranes, was done following the electric conductivity assay, as described in [117]. Then, 300 mg of mature leaf tissue was collected from each individual plant, placed in 20 mL distilled water, and shaken at 200 rpm for 2 h at room temperature, after which the initial conductivity (C1) was measured with a multi-parameter analyzer, Eijkelkamp 18.21 (Netherlands). Then, the same samples were boiled for 20 min to induce membrane disintegration and maximum electrolyte leakage, cooled to room temperature, and conductivity measured again (C2). Relative electric conductivity (%) was calculated as (C1/C2) × 100. 

### 4.6. Calorespirometric Measurements

Calorespirometric measurements were performed in the second growing year before and after plant exposure to elevated temperatures for five days (long term heat stress exposure). Measurements were done on the first expanded leaf (200–500 mg fresh weight) of five plants per mycorrhizal treatment. Two repetitions were done on the same leaf per plant and the average was used for the statistical analysis. Measurements were done in a Multi-Cell Differential Scanning Calorimeter (MC-DSC, TA Instruments, New Castle, DE, USA). Calorespirometric parameters were measured in the dark in sealed Hastelloy ampules.

First, measurements were taken at 25 °C and then, calorespirometric parameters were recorded after a heat shock of two hours at 40 °C. The same procedure was repeated again five days later, when plants had been exposed to a 40/35 °C day/night temperature regime.

At each temperature, 100–200 mg of tissue was placed in a 1 mL ampoule and, after the heat rate was constant for 5 min, the value was recorded. Then, the ampoule was opened and a small vial of 0.4 M NaOH solution was introduced, and the ampoule was closed again. The heat rate was again recorded until it was constant for 5 min. Then, the ampoule was opened, the NaOH vial was removed, and heat rate measured again until it was constant for 5 min. The CO_2_ emitted by the tissue inside the ampoule reacted with the NaOH, producing carbonate in an exothermic reaction (∆H = −108.5 kJ/mol CO_2_). Respiratory heat rates (R_q_) were calculated as the average of the first and third measurements. The CO_2_ production rate (R_co2_) was calculated from the increase in the measured heat rate during the second measurement [43]. These rates were, then, normalized to the tissue dry mass to obtain mass specific rates.

Specific growth rate or structural biomass formation rate (R_biomass_) and carbon use efficiency (Ɛ) were calculated from R_q_ and R_co2_ data with an enthalpy balance model [43,118,119] Equation (1):

(1) $$R_{q}=-\Delta H_{\mathrm{CO}2}R_{\mathrm{CO}2}-\Delta H_{\mathrm{biomass}}R_{\mathrm{biomass}}$$

Equation (1) is based on the overall reaction for plant growth from photosynthates, i.e., carbon substrates, C_S_. ΔH_CO2_ is the enthalpy change for the catabolic reactions producing CO_2_ and ΔH_biomass_ is the enthalpy change for anabolic reactions producing new structural biomass, i.e., C_biomass_ (see [51] for a detailed derivation). ΔH_CO2_ is estimated as −470 kJ/mole CO_2_ for aqueous glucose and ΔH_biomass_ was obtained from [51] and assumed to be 30 kJ/Cmole. Structural biomass formation rate, R_biomass_, can then be obtained from Equation (1). The equation for ε is obtained directly from Equation (2) and is evaluated from the ratio R_biomass_/R_CO2_ (Equation (3)):

(2) $$C_{s}+{\mathrm{xO}}_{2}\to {\varepsilon C}_{\mathrm{biomass}}+\left(1-\varepsilon \right){\mathrm{CO}}_{2}$$

(3) $$\frac{Ɛ}{1-Ɛ}=\frac{R_{\mathrm{biomass}}}{R_{\mathrm{CO}2}}$$

Data collected at 25 and 40 °C, over two hours, are indicative of the short-term heat stress response. Long-term heat stress response was determined from measurements before and after exposing plants for five days to the heat stress regime of 40/35 °C day/night, and by comparing the four calorespirometric parameters, R_q_, R_co2_ R_biomass_ and ε, at both time points and at both temperatures.

### 4.7. Near-Infrared Spectroscopy

NIR spectra of ten mature leaves per plant were collected before and after the long-term heat stress treatment (five days exposure to 40/35 °C day/night) with a Luminar 5030 Hand-held AOTF-NIR Analyzer (Brimrose Corp, MD, USA) operating in the range of 1100–2300 nm with a spectral resolution of 1 nm (a total of 1200 data points per spectrum). An aperture of 2X signal intensity was used. All spectra were returned by the device using Acquire software in reflectance mode, and were converted to absorbance as (log (1/R)) after acquisition.

### 4.8. Statistical Analysis

In the first growing year, root mycorrhizal colonization rate and total plant leaf area were analyzed by a one-way ANOVA with fixed effects, and group means were compared by Duncan’s *a posteriori* test. Normalized difference vegetation index (NDVI) was analyzed by a repeated measures analysis, as measurements were taken on the same plant along the time. A linear model was fitted, considering the following two fixed effects factors: (1) mycorrhizal inoculation, with three levels, i.e., *R. irregulare* inoculation, *F. mosseae* inoculation, and non-inoculation and (2) time, with three levels (June, August, and September) and the interaction between both factors.

In the second growing year, because measurements were taken on the same plant before and after different temperature treatments, data from the heat stress experiment were also analyzed by a repeated measures analysis. For EL, g_s_, ChlC, and Fv/Fm data, a linear model was fitted, considering the following two fixed effects factors: (1) mycorrhizal inoculation, with three levels, i.e., *R. irregulare* inoculation, *F. mosseae* inoculation, and non-inoculation and (2) long-term heat stress exposure with two levels, i.e., before and after five days heat stress exposure, as well as the interaction between both factors. The vector of random errors was assumed with multivariate normal distribution, with the vector of expected values being zero and covariance matrix assuming independence between observations from different plants and correlation among observations from the same plant exposed to the different temperature treatments.

For the analysis of calorespirometric data, another linear model was fitted with the following three fixed effects as factors: (1) mycorrhizal inoculation, with three levels, i.e., *R. irregulare* inoculation, *F. mosseae* inoculation, and non-inoculation; (2) long-term heat stress exposure with two levels, before and after five days heat stress exposure; and (3) short-term heat stress exposure, with two levels, i.e., non-stress (2 h at 25 °C) and heat shock (2 h at 40 °C), and their respective interactions. The same assumptions previously described for the vector of random errors were used, including a covariance matrix, which allowed for correlation among observations from the same plant exposed to different treatments. 

In all cases, after the repeated measures analyses, pairwise multiple comparisons of means were performed using the simulation method of Edwards and Berry [70]. Data analysis was performed in SAS version 9.4 (SAS Institute Inc., Cary, NC, USA) using MIXED procedure.

To analyze the NIR spectral data, spectra were first subjected to a standard normal variate pretreatment in the 1100–2300 nm range, after which the average spectra of 10 leaves per plant was calculated and a matrix was created, where the rows corresponded to the average spectra of each plant and the columns to each wavelength (from 1100 to 2300 nm). An unsupervised pattern recognition technique, principal component analysis (PCA), was carried out prior to quantitative analysis to provide information about the latent structure of the spectral matrix [71,120] and to detect outliers. Then, this same matrix was used as calibration matrix by adding the corresponding reference values of each sample, i.e., g_s_, EL, ChlC, Fv/Fm, R_q_, R_co2_, R_biomass_, and Ɛ. These were set as dependent variables (Y), while spectral data were set as the independent (X) variables. Next, calibrations were performed by modified partial least squares (MPLS) regression. Scattering effects were removed using multiplicative scatter correction (MSC), standard normal variate (SNV), or detrending [121,122]. In the MPLS regression method, the spectral matrix is divided into a series of subsets in order to perform a cross-validation procedure, to set the number of PLS factors, to reduce the possibility of overfitting, and to remove chemical outliers [71]. Using the T ≥ 2.5 criterion, samples that presented a high residual value when they were predicted were eliminated from the set. Finally, validation errors were combined into a single parameter, i.e., the standard error of cross-validation (SECV). A leave-one-out cross-validation approach was used in the present study. The leave-one-out approach is a special case of the k-fold cross-validation method, a well-established method that segments the data into k partitions, where k = number of samples [121,122]. Furthermore, the effect of differentiation and variations in spectral ranges were tested in the development of the NIR spectral calibrations. The software used was Win ISI^®^ (v1.50) (Infrasoft International, LLC, Port Matilda, PA, USA).

## 5. Conclusions

A major challenge to phenotyping heat stress tolerance is the selection of suitable phenotypic traits to measure. The calorespirometric parameters R_biomass_ and Ɛ showed the highest accuracy for assessing both short- and long-term heat stress responses. Among the commonly measured plant physiology traits affected by high temperatures, g_s_ was the only measure that detected differences in heat tolerance among the three mycorrhizal inoculation treatments. Because heat stress affects young and older tissues differently, and calorespirometric measurements are only effective when done in actively growing tissues, determining the effect of high temperatures on the physiology of older organs requires different measurements. We propose the combination of calorespirometric measurements on young growing tissues with gas exchange measurements and NIR measurements on mature tissues as a useful technique for determining heat tolerance in newly developed breeding material under controlled conditions, and thus to accurately select new grapevine clones to be tested under field conditions.

## Figures and Tables

**Table 1:** Root mycorrhizal colonization rate three months after inoculation (*n* = 3) and ten months after inoculation (*n* = 5) in ‘Touriga Nacional’ variety vines grafted onto 1103 Paulsen rootstock. Data represent the average values ± standard error. Different letters indicate significant differences in group means for each time point according to Duncan’s *a posteori* test. Significant effect, *p*-value < 0.05.

Inoculation Treatment	3 Months after Inoculation	10 Months after Inoculation
Non-inoculated	0.78 ± 0.007 a	0.81 ± 0.296 a
Inoculated with *R. irregulare*	0.63 ± 0.008 b	0.75 ± 0.164 b
Inoculated wit*h F. mosseae*	0.70 ± 0.039 ab	0.76 ± 0.379 ab

**Table 2:** *P*-values of the test for the analysis of the effects of the different factors and their interactions. Statistical analysis was done by fitting a linear model considering two fixed factors (mycorrhizal inoculation and long-term heat stress exposure), their interaction, and an error covariance matrix allowing correlation among observations from the same plant exposed to the different treatments. Significant effect, *p*-value < 0.05.

Effects	*p*-Value			
	Relative Electrolite Leakage	Stomatal Conductance	Ratio of Variable to Maximum Chlorophyll Fluorescence	Relative Chlorophyll Content
Long-term heat stress exposure	0.004	<0.001	0.325	<0.001
Mycorrhizal inoculation	0.638	0.006	0.959	0.675
Long-term heat stress exposure x Mycorrhizal inoculation	0.276	0.061	0.907	0.586

**Table 3:** Measurements of physiological parameters in plants inoculated or not with mycorrhizal fungi and exposed or not to a long-term heat stress treatment. Values indicate mean ± standard error (n = 5). Different letters indicate significant differences in group means according to pairwise multiple comparisons conducted using the simulation method of Edwards and Berry [70]. Significant effect, *p*-value < 0.05.

Mycorrhizal Treaments	Relative Electrolite Leakage (%)		Stomatal Conductance (mmol. m^2^.s^−1^)		Ratio of Variable to Maximum Chlorophyll Fluorescence (Ratio)		Relative Chlorophyll Content (SPAD Units)	
	Before Stress	After 5-Day Exposure to Heat Stress	Before Stress	After 5-Day Exposure to Heat stress	Before Stress	After 5-Day Exposure to Heat Stress	Before Stress	After 5-Day Exposure to Heat Stress
Non-inoculated	4.51 ± 0.438	3.63 ± 0.121	67 ± 8.5 b	119 ± 19.6 ab	0.7587 ± 0.0046	0.7579 ± 0.0047	4.24 ± 0.327	5.47 ± 0.282
Inoculated with *R. irregulare*	5.11 ± 0.583	3.81 ± 0.255	58 ± 1.8 b	228 ± 29.0 a	0.7625 ± 0.0077	0.7574 ± 0.0031	4.32 ± 0.186	5.91 ± 0.303
Inoculated with *F. mosseae*	4.21 ± 0.219	3.90 ± 0.160	65 ± 7.8 b	190 ± 8.8 a	0.7606 ± 0.0046	0.7552 ± 0.0046	4.69 ± 0.259	5.67 ± 0.478

**Table 4:** *P*-values of the test for the effects of the different factors and their interactions. Statistical analysis was done by fitting a linear model considering three fixed factors (mycorrhizal inoculation short-term heat stress and long-term heat stress), their interaction, and an error covariance matrix allowing correlation among observations from the same plant exposed to the different treatments. Significant effect, *p*-value < 0.05.

Effects	*p*-Value			
	Metabolic Heat Rate (R_q_)	Respiratory Rate (R_co2_)	Structural Biomass Formation Rate (R_biomass_)	Carbon use Efficiency (Ɛ)
Long-term heat stress exposure	<0.0001	<0.0001	<0.0001	0.0003
Short-term heat stress exposure	<0.0001	0.3799	<0.0001	<0.0001
Mycorrhizal inoculation	0.1069	0.1602	0.6960	0.4647
Long-term heat stress exposure x mycorrhizal inoculation	0.5035	0.9855	0.8484	0.3530
Short-term heat stress exposure x mycorrhizal inoculation	0.8677	0.9479	0.6674	0.1634
Long-term heat stress exposure x short-term heat stress exposure	0.5035	0.7915	0.0556	0.9512
Long-term heat stress exposure x short-term stress x mycorrhizal inoculation	0.2361	0.7279	0.8596	0.7685

**Table 5:** Main statistical descriptors for the modified partial least squares (PLS) models developed in the near infrared reflectance zone close to 1100–2300 nm.

Spectral Pre-Treatments *^a^*	Reference Variables	N *^b^*	PLS Factors	Mean	SD *^c^*	SEC *^d^*	RSQ *^e^*	SECV *^f^*	SECV (%)	SEP *^g^*	SEP (%)
SNV + detrend 2,5,5,1	Fv/Fm^1^	27	2	0.76	0.01	0.01	0.72	0.01	1.81	0.01	0.66
Detrend 0,0,1,1	EL^2^	25	4	3.94	0.51	0.22	0.81	0.33	8.43	0.20	5.05
SNV 2,10,10,1	ChlC^3^	28	4	5.05	0.98	0.30	0.91	0.67	13.37	0.27	5.35
MSC 0,0,1,1	Ɛ^4^	16	3	0.66	0.17	0.10	0.67	0.14	20.78	0.08	12.60
SNV + detrend 0,0,1,1	R_q_^5^	25	2	5343.0	1530.57	1117.66	0.47	1229.66	23.01	1048.46	19.62
SNV 2,5,5,1	R_co2_^6^	28	3	12.02	6.19	2.37	0.85	4.76	31.14	2.20	14.38
MSC 2,10,10,1	g_s_^7^	27	1	112.37	62.92	49.14	0.39	56.83	37.74	47.29	31.41
MSC 0,0,1,1	R_biomass_^8^	16	3	45.69	26.65	16.21	0.63	24.39	38.82	14.04	22.34

^1^ Ratio of variable fluorescence and maximum fluorescence, unitless; ^2^ relative electrolyte leakage, in percentage units; ^3^ relative chlorophyll content, in SPAD relative units; ^4^ carbon use efficiency, ratio; ^5^ metabolic heat rate, in mJ s^−1^ g^−1^ dry weight; ^6^ respiratory rate, in nmol CO_2_ s^−1^ g^−1^ dry weight; ^7^ stomatal conductance, in mmol m^2^ s^−1^; ^8^ structural biomass formation rate, in nmol C s^−1^ g^−1^ dry weight. *^a^*Spectral pretreatments are denoted by four numbers, i.e., first number, the number of the derivative; second number, gap over which the derivative is calculated; third number, the number of data points in a running average or smoothing; and fourth number, second smoothing according to [71]. *^b^*N, number of samples used to obtain the calibration equation after eliminating samples for chemical reasons (T criterion); *^c^*SD, standard deviation; *^d^*SEC, standard error of calibration; *^e^*RSQ, coefficient of determination; *^f^*SECV, standard error of cross-validation (8 cross-validation groups); *^g^*SEP, standard error of prediction (internal validation).
